# Rapid Suppression of Activated Rac1 by Cadherins and Nectins during *De Novo* Cell-Cell Adhesion

**DOI:** 10.1371/journal.pone.0017841

**Published:** 2011-03-11

**Authors:** Khameeka N. Kitt, W. James Nelson

**Affiliations:** 1 Department of Biology, The James H. Clark Center, The Bio-X Program, Stanford University, Stanford, California, United States of America; 2 Department of Molecular and Cellular Physiology, The James H. Clark Center, The Bio-X Program, Stanford University, Stanford, California, United States of America; Leiden University, Netherlands

## Abstract

Cell-cell adhesion in simple epithelia involves the engagement of E-cadherin and nectins, and the reorganization of the actin cytoskeleton and membrane dynamics by Rho GTPases, particularly Rac1. However, it remains unclear whether E-cadherin and nectins up-regulate, maintain or suppress Rac1 activity during cell-cell adhesion. Roles for Rho GTPases are complicated by cell spreading and integrin-based adhesions to the extracellular matrix that occur concurrently with cell-cell adhesion, and which also require Rho GTPases. Here, we designed a simple approach to examine Rac1 activity upon cell-cell adhesion by MDCK epithelial cells, without cell spreading or integrin-based adhesion. Upon initiation of cell-cell contact in 3-D cell aggregates, we observed an initial peak of Rac1 activity that rapidly decreased by ∼66% within 5 minutes, and further decreased to a low baseline level after 30 minutes. Inhibition of E-cadherin engagement with DECMA-1 Fab fragments or competitive binding of soluble E-cadherin, or nectin2alpha extracellular domain completely inhibited Rac1 activity. These results indicate that cadherins and nectins cooperate to induce and then rapidly suppress Rac1 activity during initial cell-cell adhesion, which may be important in inhibiting the migratory cell phenotype and allowing the establishment of initially weak cell-cell adhesions.

## Introduction

Cell-cell adhesion is essential for the development and maintenance of tissue structure and function, and is mediated by different classes of junctional membrane proteins, principally cadherins and nectins [1], [2]. Cadherins are the major class of calcium-dependent transmembrane proteins involved in initial cell-cell adhesion. For cell-cell adhesions to be established and maintained, cadherins undergo weak *trans* homophilic interactions, which promote lateral clustering of additional cadherin molecules to strengthen cell-cell contacts [3], [4], [5]. Nectins are calcium-independent immunoglobulin-like cell-cell adhesion molecules that form homo-*cis* dimers and hetero-*trans* dimers via their extracellular domains and associate with the cytoskeleton through the F-actin binding protein, afadin [6], [7]. Nectins participate in cell-cell adhesion by interacting with E-cadherin via their cytoplasmic domain-associated proteins [8], and by regulating E-cadherin *trans*-interactions at cell-cell contacts [9], [10], [11].

The actin cytoskeleton plays a major role in regulating cell shape, cell migration and plasma membrane dynamics [12], [13], [14]. The Rho GTPases, RhoA, Rac1 and Cdc42, are a class of proteins that are key regulators of the actin cytoskeleton in coordinating cell migration and cell-cell adhesion [15], [16]. Rho GTPases are cytosolic proteins that cycle between two different conformations depending on extracellular or intracellular stimuli: an active GTP-bound state and an inactive GDP-bound state. The analysis of Rho GTPases during initial cell-cell adhesion between migrating cells is complicated by two additional dynamic cellular processes, cell spreading and integrin-based adhesion to the extracellular matrix both of which are also regulated by Rho GTPases. As a result, analysis of Rho GTPases activity in traditional adhesion assays in 2-D has given rise to apparently contradictory conclusions.

Biochemical studies of Rac1 activity levels in whole cell populations at different times after initiation of cell-cell contacts found that Rac1 activity increased, and was subsequently maintained at a high level upon E-cadherin adhesion [17], [18]. Using a different assay involving Chinese Hamster Ovary (CHO) cells spreading on a substratum of C-cadherin, Noren et al also reported a gradual increase in Rac1 activity that reached a peak at 1 hour, analogous to results from cell-cell adhesion in 2-D cultures of cells spreading on a plastic surface. Nakagawa et al [19] reported a similar trend in Rac1 activation in MDCK cells. These results indicate that E-cadherin – mediated cell-cell adhesion initiates and then sustains high Rac1 activity.

Live-cell imaging of Rac1 localization and activity levels provides a direct approach to examine the localization of Rac1 activation. Using a laser trap to place small beads coated with the extracellular domain of E-cadherin on a cell surface to initiate E-cadherin adhesion, Perez et al reported a very transient increase (<5 minutes) in cellular Rac1 localization around the bead [20]. In a separate study, direct examination of Rac1 activity during initial cell-cell adhesion using the Raichu-Rac1/FRET biosensor revealed active Rac1 at the edges of expanding cell-cell contacts, but relatively low levels at existing cell-cell contacts. These observations, in contrast to biochemical using whole cell population measurements of Rac1 activity, indicate that upon E-cadherin-mediated cell-cell adhesion Rac1 activity is initially increased, and is then rapidly down-regulated [21].

Activation of Rac1 during initial cell-cell adhesion is also dependent on the *trans*-interaction of nectins [22], [23], [24]. Furthermore, nectins may regulate the formation of cell-cell contacts in cooperation with E-cadherin [8], [10], [11]. Recombinant nectin extracellular domain has both stimulatory and inhibitory effects on E-cadherin recruitment to cell membranes or coated beads [9], [10], [23]. Although nectin engagement is able to cause a gradual increase in Rac1, the role of nectins in regulating Rac1 activity with E-cadherin at cell-cell contacts remains poorly understood.

Taken together, these studies raise the question whether E-cadherin and/or nectin engagement activates or suppresses Rac1 activity during initial cell-cell adhesion. Different experimental conditions may have lead to different Rac1 activity profiles and conclusions. Cell spreading and integrin-based adhesion to the extracellular matrix are both known to require activation of Rho GTPases that would effect the analysis of Rac1 in whole cell assays [25], [26]. Therefore, we sought to develop whole cell assays of Rac1 activity during cell-cell adhesion under conditions that eliminated cell spreading and integrin-based adhesion. Our results indicate that an initial high level of Rac1 activity is rapidly suppressed within 5 minutes of Ca^++^-dependent adhesion, and that both E-cadherin and nectins are involved.

## Results

**Figure 1:**
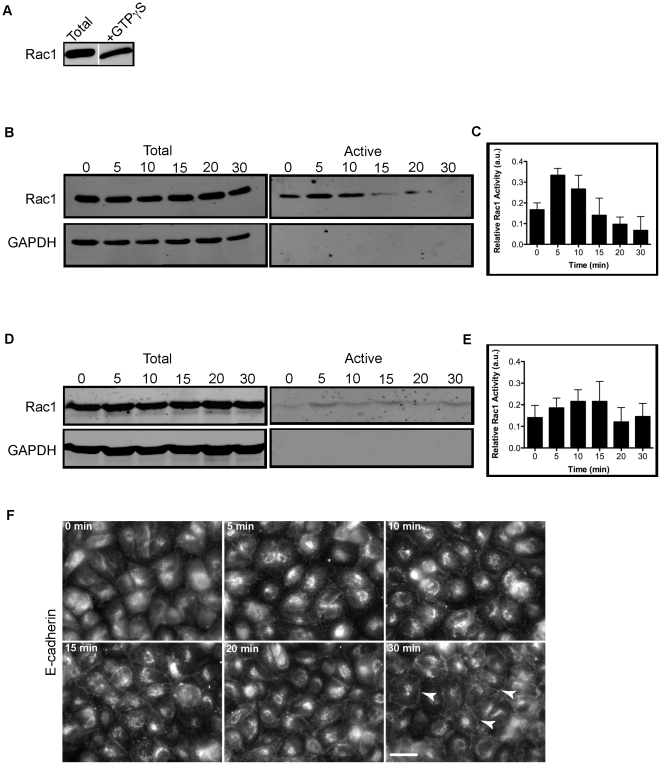
Transient increase, then decrease in Rac1 activity upon cell-cell adhesion. (A) GST-PAK domain pulls down high levels of active Rac1 in the presence of GTPgammaS. Cell-cell adhesion in whole cell populations was induced in contact naïve MDCK GII cells (as described in Experimental Procedures) plated at confluence on uncoated tissue culture plastic (B) or collagen-I coated tissue culture plastic plates (D). Cell lysates for each time point were incubated with GST-PAK and separated by SDS-PAGE followed by immunoblotting for Rac1 and GAPDH. Under both conditions, there is a gradual increase, followed by a decrease in Rac1 activation upon induction of cell-cell contact. Bar graphs show quantification of Rac1-GTP (activation) on tissue culture plastic (C) or collagen-I coated plates (E). Error bars are SEM of 3 (C) and 4 (E) independent experiments. Endogenous E-cadherin protein gradually increases and redistributes to cell-cell contacts by 30 minutes (arrows) (F). Scale bar: 10 µm.

We sought to test different experimental conditions to examine cell-cell adhesion and Rac1 activity in a whole cell population assay. We used GST-PAK (p21-activating kinase) to isolate active Rac1^GTP^ from cell lysates at different times after induction of cell-cell adhesion. We first confirmed that GST-PAK protein is able to pull down active Rac1. MDCK cell lysates were loaded with GTPgammaS and a Rac1 activation assay was performed, resulting in the isolation of high levels of active Rac1 (Figure 1A).

We first used a traditional assay to measure basal levels of active Rac1 during cell-cell adhesion. MDCK cells were allowed to adhere for 3 hours to uncoated tissue culture plastic in medium containing 5 µM calcium to block Ca^++^-dependent cell-cell adhesion. Upon restoration of calcium levels to 1.8 mM, there was a rapid increase in Rac1 activity between 0 and 5 minutes followed by a gradual decrease over 25 minutes to base-line levels at 30 minutes (Figure 1, B and C). When MDCK cells were allowed to adhere to collagen I-coated tissue culture plates for 3 hours and induced to form cell-cell contacts, there was a gradual increase in Rac1 activity from 0 to 15 minutes followed by a decrease to baseline levels at 30 minutes (Figure 1, D and E). No statistical significant difference in Rac1 activity was observed between cells plated on plastic vs. collagen I-coated plates. In these and in all subsequent experiments, cells were kept in normal serum concentration (10%) at all times to ensure the effects on Rac1 activation were due to a change in Ca^++^ concentration levels and engagement of cell-cell adhesion proteins. To determine whether the changes in Rac1 activity corresponded to a change in the localization of E-cadherin upon calcium switch, cells plated on collagen-coated coverslips were fixed and analyzed by immunofluorescence microscopy. Epifluorescence imaging showed a gradual increase in endogenous E-cadherin protein at cell-cell contacts between 5–10 minutes, to clear localization at cell-cell contacts by 30 minutes (Figure 1F, arrowheads) [27]. Similar results are expected if cells were plated on uncoated coverslips. Taken together, these results show a transient increase, then decrease in Rac1 activity upon cell-cell adhesion, cell spreading and integrin-based adhesion.

**Figure 2:**
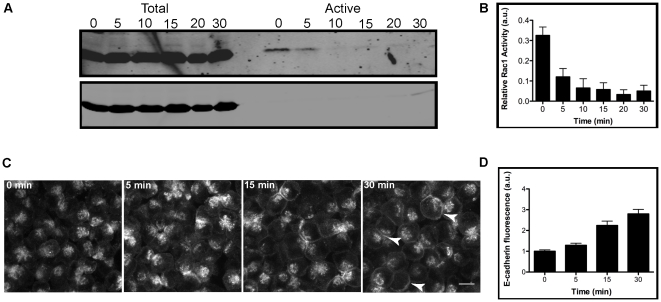
Rac1 activity rapidly decreases upon cell-cell adhesion in 3-D cell aggregates. Contact naïve MDCK GII cells were trypsinized, resuspended in low calcium media, and spun down to form 3-D cell aggregates. Cell-cell adhesion was induced by incubating cells in normal calcium media and cell lysates for each time point were incubated with GST-PAK and separated by SDS-PAGE followed by immunoblotting for Rac1 and GAPDH. High levels of active Rac1 at 0 minutes rapidly decreased (∼66%) within 5 minutes of cell-cell adhesion, and declined to a baseline level by 30 minutes (A). Bar graph shows quantification of Rac1 activation (B); error bars are SEM of 4 independent experiments. 3-D E-cadherin-RFP MDCK stable cell aggregates were fixed and imaged (C). E-cadherin-RFP protein gradually increases and redistributes to cell-cell contacts by 30 minutes (arrows). Bar graph shows quantification of E-cadherin fluorescence intensity at cell-cell contacts (D); error bars are SEM of 50 independent cell-cell contacts. Bar: 10 µm.

To isolate the role of cell-cell contact on Rac1 activity, in the absence of cell spreading or integrin-based adhesion, cell-cell contacts were rapidly induced by pelleting a cell suspension into a 3-D cell aggregate. Contact naïve MDCK GII cells were trypsinized, resuspended in low calcium media (5 µM), counted, and centrifuged in low calcium media (t = 0 minutes) or normal calcium media (1.8 mM) (t = 5–30 minutes) to form a 3-D cell aggregate pellet. To induce cell-cell adhesion, cell aggregates were in incubated for the indicated times. Note that the very short time-frame that cells were in suspension without cell-cell contact was much less than the time required for detached cells to undergo anoikis [28]; in addition, DAPI staining of cell nuclei during the time course of cell aggregation did not reveal evidence of apoptosis (KK, unpublished results). The level of Rac1 activity was initially high (time = 0, taken as the time immediately after centrifugation). However, within 5 minutes of cell-cell adhesion, the level had decreased by ∼66% and further declined to a baseline level by 30 minutes (Figure 2, A and B). This initial high level and rapid decrease in Rac1 activity was not observed if cells were maintained in 5 µM Ca^++^ to inhibit cadherin-mediated cell-cell adhesion (KK, unpublished results).

To determine if the changes in Rac1 active levels coincided with changes in the distribution of E-cadherin, 3-D MDCK cell aggregates stably expressing E-cadherin-RFP were fixed and analyzed by immunofluorescence microscopy. We used E-cadherin-RFP cells due to the variable penetrance of the E-cadherin antibody into the 3-D aggregates. Addition of RFP to E-cadherin does not disrupt normal localization of E-cadherin protein to cell-cell contacts (KNK, unpublished results) [20], [29]. Laser-scanning, confocal imaging revealed a gradual redistribution of diffuse staining of E-cadherin-RFP at time  = 0, to clear localization along cell-cell contacts by 30 minutes (Figure 2, C [arrowheads] and D). These results indicate that the recruitment of E-cadherin to cell-cell contacts coincides with the regulation of Rac1 activity upon Ca^++^ - dependent cell-cell adhesion.

**Figure 3:**
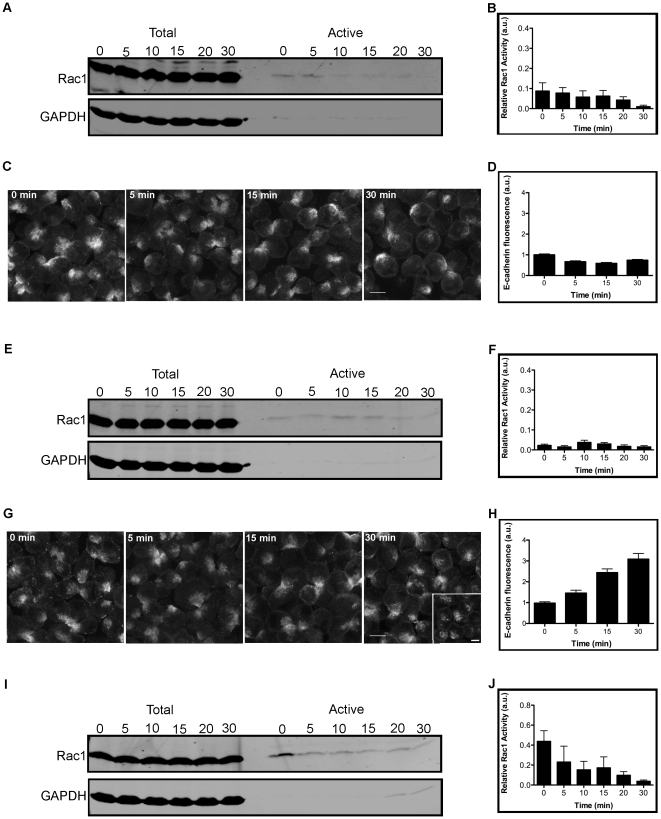
Rac1 activity decreases in the presence of DECMA-1 and E-cadherin:Fc in 3-D cell aggregates. 3-D MDCK cell aggregates were incubated with 10 µg/ml of DECMA-1 monovalent Fab' fragments (A), or 10 µg/ml of purified E-cadherin:Fc extracellular domain (E), or 10 µg/ml of Fc protein (I) for the indicated times. Cell lysates for each time point were incubated with GST-PAK and separated by SDS-PAGE followed by immunoblotting for Rac1 and GAPDH. Blocking E-cadherin or addition of exogenous E-cadherin:Fc decreased Rac1 activity levels. Fc protein did not affect Rac1 active levels. Bar graph shows quantification of Rac1 activation for DECMA-1 monovalent Fab' fragments (B) or E-cadherin:Fc (F) or Fc protein (J); error bars are SEM of 4 independent experiments for each condition. Incubation of cell aggregates with the Fab' fragments inhibited E-cadherin recruitment to cell-cell contacts over 30 minutes (C); addition of E-cadherin:Fc caused patchy recruitment of cellular E-cadherin to the cell surface by 30 minutes, compared to compacted cell-cell contacts under control conditions (see inset-30 min) (G). Bar graphs show quantification of E-cadherin fluorescence intensity at cell-cell contacts for DECMA-1 Fab' fragments (D) or E-cadherin:Fc; error bars are SEM of 50 independent cell-cell contacts. Bars: 10 µm and 5 µm.

We tested whether E-cadherin engagement was required for the regulation of Rac1 activity in 3-D aggregates; note that MDCK cells also express K-cadherin (Cadherin-6), but we did not examine its role in regulating Rac1 activity due to its low expression in single cells [30] and that its depletion does not significantly disrupt the establishment and maintenance of cell organization [31]. Although E-cadherin expression could be depleted by siRNA, this method does not directly isolate the functional properties of the protein. To examine in real-time whether inhibition of E-cadherin engagement between cells affects Rac1 activity, we employed two approaches. First, cell suspensions were incubated with monovalent Fab' fragments of the E-cadherin blocking antibody DECMA-1 [32], and then the cells were pelleted to form 3-D aggregates (as described above for control conditions). Inhibition of E-cadherin engagement resulted in a low baseline level of Rac1 activity over 30 minutes (Figure 3, A and B). This low baseline level of Rac1 activity observed at time 0 in the presence of Decma Fab' fragments is statistically lower than the level of Rac1 activity in control conditions (see Figure S1, A and B). To confirm that the DECMA-1 Fab' fragments inhibited E-cadherin redistribution and cell-cell adhesion, 3-D MDCK cell aggregates stably expressing E-cadherin-RFP were incubated with DECMA-1 Fab' fragments, fixed and imaged. This analysis revealed that E-cadherin-RFP remained diffusely distributed and that there was little to no recruitment of E-cadherin to cell-cell contacts by 30 minutes (Figure 3, C and D), in contrast to control cells (Figure 2, C and D). These results indicate that changes in Rac1 activity during initial cell-cell contact are dependent on E-cadherin engagement and reorganization to cell-cell contacts.

Second, we tested whether direct competition of E-cadherin – based cell-cell adhesion by exogenous E-cadherin extracellular domain affected Rac1 activity in 3-D cell aggregates. A purified chimeric protein comprised of the extracellular domain of E-cadherin fused at its C-terminus to the Fc domain of human IgG1 (E-cadherin:Fc) [33] was added to cell suspensions, and the cells were then pelleted to form 3-D cell aggregates. We detected little to no Rac1 activity over 30 minutes (Figure 3, E and F). Again, the low baseline level of Rac1 activity observed at time 0 for E-cadherin:Fc is statistically lower than the level of Rac1 activity in control conditions (see Figure S1, A and B). Imaging and quantification of 3-D cell aggregates revealed a gradual increase in E-cadherin-RFP at the plasma membrane (Figure 3, G and H), but the contacts between cells were less compacted and the E-cadherin-RFP was patchy and not uniform, compared to control conditions (inset Figure 3G) suggesting some aggregation of cellular E-cadherin by E-cadherin:Fc, but little or no cell-cell adhesion. The Fc domain alone did not affect changes in Rac1 active levels, which were similar to controls (Figure 3, I and J).

**Figure 4:**
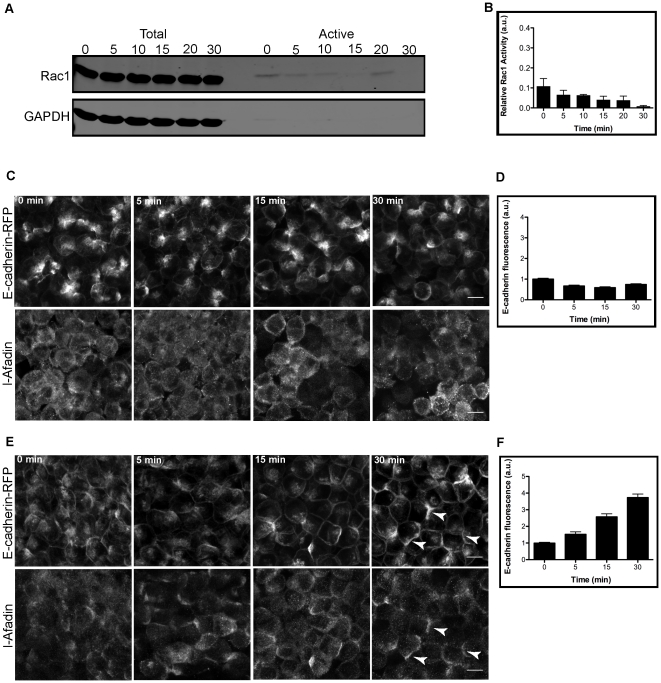
Decrease in Rac1 activity in the presence of nectin2alpha:Fc in 3-D cell aggregates. 3-D MDCK cell aggregates were induced to form cell-cell contacts in the presence of 10 µg/ml of purified nectin2alpha:Fc extracellular domain for the indicated time points. Cell lysates were incubated with GST-PAK and separated by SDS-PAGE followed by immunoblotting for Rac1 and GAPDH. Inhibition of nectin *trans*-interactions caused little to no Rac1 activity over 30 minutes (A). Bar graph shows quantification of Rac1 activation (B); error bars are SEM of 4 independent experiments. 3-D E-cadherin-RFP MDCK stable cell aggregates were fixed, labeled for endogenous afadin, and imaged (C). E-cadherin-RFP recruitment to cell-cell contacts peaked by 15 minutes with diffuse localization of afadin at cell-cell adhesions in the presence of nectin2alpha:Fc (D). E-cadherin-RFP is recruited to cell-cell contacts along with afadin in the absence of nectin2alpha:Fc (arrows) (E and F). Bar graphs show quantification of E-cadherin fluorescence intensity at cell-cell contacts (D and F); error bars are SEM of 50 independent cell-cell contacts. Bar: 10 µm.

We tested the role of nectins in Rac1 activation and subsequent inactivation during *de novo* cell-cell adhesion. To competitively inhibit nectin *trans*-interaction, we used a purified chimeric protein comprised of the extracellular domain of nectin2alpha fused at its C-terminus to the Fc domain of human IgG1 (nectin2alpha:Fc) [34]. nectin2alpha:Fc was added to cell suspensions, which were then pelleted to form 3-D cell aggregates. There was little to no Rac1 activity over 30 minutes (Figure 4, A and B). Statistical comparison of Rac1 activity at time 0 of nectin2alpha:Fc and control cells showed a significant decrease (see Figure S1, A and B). Because endogenous nectin levels are very low in MDCK cell, we labeled cells for afadin as a surrogate for nectin localization, since afadin binds to the cytoplasmic domain of nectin [35]. We observe diffuse labeling of afadin with little or no localization at cell-cell contacts (Figure 4, C and D). In the absence of nectin2alpha:Fc, we observe normal recruitment of E-cadherin and afadin to cell-cell contacts (arrowheads) by 30 minutes (Figure 4, E and F). Due to the variable penetrance of the afadin antibody into fixed 3-D cell aggregates, afadin fluorescence at cell-cell contacts were not measured. Imaging and quantitative analysis of 3-D cell aggregates showed a gradual increase in E-cadherin-RFP recruitment to cell-cell contacts that peaked after 15 minutes (Figure 4, C and D). However, the amount of E-cadherin at cell-cell contacts appeared to be lower than under control conditions (Figure 4, E and F), as reported previously under these conditions [9], [10], [11]. These results indicate that nectins are also involved in regulating Rac1 activity during initial cell-cell adhesion.

## Discussion

Previous studies reported a gradual increase in Rac1 activity upon cell-cell adhesion [19], [23], [24], [36], [37]. However, in those studies cells not only made contact with neighboring cells but were also actively spreading on a substrate (extracellular matrix, or C-cadherin:Fc–coated surface). Cell spreading alone has been shown to increase Rac1 activity [38]. Indeed, our initial studies confirmed a gradual increase in Rac1 activity upon cell-cell adhesion in cells spreading on a substratum in the presence or absence of collagen I, similar to published results [19], [37]. Therefore, the observed effects of cadherin engagement on increasing Rac1 activity might not be due directly to cadherin binding, but could depend on additional cellular processes including integrin engagement and cell spreading. Integrin engagement and adhesion to the extracellular matrix has an effect on the activation of Cdc42 and Rac1 [39], [40], [41]. However, this effect is mediated by cellular spreading on the extracellular matrix [41]. Thus, the goal of this study was to isolate the role of cadherins on GTPase activation during initial cell-cell adhesion without interference from integrin-extracellular adhesion.

To circumvent these complications, we devised a simple method to isolate the role of cell-cell adhesion on Rac1 activity. We focused on the dynamics of Rac1 activation because Cdc42 activation was not observed under similar conditions (KNK, unpublished results), and Rac1 activation has been shown to be involved in simple epithelial (MDCK) cell-cell adhesion [17], [42]. To examine only cell-cell adhesion, cells were pelleted into a 3-D cell aggregate to force the cells to make contact with one another without spreading or engagement of integrins. Although the cells are detached from their substrate, the time course used in the activation assays was less than the time required for the cells to undergo anoikis (3–5 hours after detachment [28]). Furthermore, each assay condition was initiated 35 minutes after trypsinizaton, E-cadherin protein levels were sufficient to induce cell-cell adhesion before activation assays were performed (see Figure 2C, time 0). We found an initial high level of Rac1 activity, which decreased rapidly within 5 minutes of Ca^++^-dependent cell-cell adhesion. These results are different than other whole population studies of Rac1 activity during cell-cell adhesion [19], [36], [37]. However, we propose that those previous studies necessarily included the role of integrin-based adhesion and cell spreading in cell-cell adhesion assays performed in 2-D.

In untreated, control cells, we observed a high level of active Rac1 at the 0 min time point, before the switch to 1.8 mM Ca^++^. It is possible that a fraction of E-cadherin, even in the presence of 5 µM Ca^++^ can induce Rac1 activation. Indeed, a recent study found monomeric cadherin *trans*-interactions in the presence of EGTA that gradually decreased over time, indicating that a small fraction of cadherins bound Ca^++^ to adopt a proper conformation for *trans*-interactions until an equilibrium was reached [5]. Significantly, this initial high level of Rac1 was inhibited by blocking E-cadherin adhesion with DECMA-1 Fab fragments (see also [19], [37]), or by competing E-cadherin *trans*-interactions with the extracellular domain of E-cadherin (see Figure S1).

Addition of nectin2alpha:Fc also inhibited Rac1 activity in 3-D cell aggregates. Recently, Sato et al examined the effect of afadin depletion on E-cadherin recruitment to nectin-based cell-cell contacts [10]. A non-*trans*-interacting form of E-cadherin was found to assemble at the membrane, which then formed *trans*-interactions stimulated by nectin and afadin. Hence, E-cadherin:Fc and nectin2alpha:Fc may have had similar effects by inhibiting normal E-cadherin *trans*-interactions between cells, and activation of Rac1 (see Figure S1). Alternatively, E-cadherin and nectin2alpha:Fc fusion proteins may activate adhesion in the cell suspensions by binding to their respective cellular membrane proteins in the absence of calcium before the assay has begun. Taken together, these results indicate that nectins and E-cadherin cooperatively activate Rac1 activity upon initial cell-cell adhesion [8], [10], [11] even in low Ca^++^.

Our assay revealed that the initial high Rac1 activity (in the presence of normal serum concentrations) was reduced by ∼66% within 5 minutes following induction of Ca^++^-dependent cell-cell adhesion. These results are different from published reports of a gradual increase in Rac1 activity upon switching cells to high serum and calcium concentrations after long periods of serum starvation. Live-cell studies of Rac1 [20] and activated Rac1 (Raichu: Rac1-GTP; [21]) protein distributions during initial E-cadherin-mediated cell-cell adhesion, also showed a rapid decrease in Rac1 and active Rac1 at newly formed cell-cell contacts. Interestingly, the loss in total Rac1 localization at newly-formed contacts occurred within 5 minutes of E-cadherin recruitment [20]. Thus, our whole population analysis of active Rac 1 in 3-D aggregates fits with these direct studies of Rac1 protein distributions in individual cells.

Our results indicate that cell-cell adhesion and signaling by E-cadherin and nectins may locally activate or recruit locally a guanine nucleotide exchange factor (GEF) to initially activate Rac1, and activate or recruit locally a GAP (GTPase activating protein) to inactivate Rac1 at cell-cell contacts. The transient activation of Rac1 by GEF(s) may be important in allowing dynamic membranes to promote contacts between cells, but the further establishment of initially weak cell-cell adhesion may require dampening of membrane dynamics by rapid down-regulation of Rac1 activity following activation of GAP(s). This may require downstream signaling events mediated by E-cadherin and nectin engagement. Indeed, a constitutively-active form of Rac1 was found to persist at initial cell-cell contacts [20], presumably because it could not be down-regulated by GAP(s). The identity of these GEFs and GAPs remains poorly understood, and are beyond the scope of the present study. However, the assay that we have developed and the identification of the time frame for activation and inactivation of Rac1 by cell-cell adhesion provide the basis for a screen to identify candidates.

## Materials and Methods

### Cell Culture

Madin-darby canine kidney (MDCK) GII cells [27] were maintained in DMEM with 1 g/L sodium bicarbonate, 10% fetal bovine serum (Atlas Biologicals), penicillin, streptomycin, and kanamycin. MDCK E-cadherin-RFP stable cell lines were generated and maintained as described [43], [44].

### Antibodies, Constructs, and Purified Proteins

E-cadherin:Fc fusion protein was generated and purified as described [33]. For generation of the nectin2alpha:Fc chimeric fusion protein (cDNA of nectin2alpha provided by Dr. Y. Takai [Osaka University, Japan]), the extracellular domain of nectin2alpha was cloned into a CDM8 expression vector containing CH2 and CH3 domains of human IgG1 (similar to E-cadherin:Fc [33]). Nectin2alpha:Fc fusion protein was purified as described [33]. DECMA-1 monovalent Fab' fragments were generated from the E-cadherin blocking antibody DECMA-1 ([32], Sigma-Aldrich) using the Fab Micro Preparation Kit from Pierce Thermo Scientific. Monoclonal E-cadherin antibody was purchased from BD Biosciences. Rabbit anti-l-afadin polyclonal antibody was purchased from Sigma-Aldrich, and GTPgammaS from Pierce Thermo Scientific. pGEXTK-PAK1 70-117 (GST-PAK) plasmid was purchased from Addgene (Addgene plasmid 12217) and provided by Jonathan Chernoff (Fox Chase Cancer Center, PA).

### Rac1 Activation Assays in 2-D

For cell substrate and integrin-based adhesions, contact naïve MDCK GII cells were trypsinized, resuspended in low calcium media (5 µM) to inhibit cell-cell adhesion, and plated at confluency (6×10^6^ cells/60 mm diameter plate) for 3 hours. At time 0, low calcium media was replaced with normal Ca^++^ (1.8 mM) media to induce cell-cell adhesion. Activated Rac1 was measured in cells at different time point. Briefly, 25 µg of purified GST-PAK (isolated from BL21-DE3 bacterial cells) was incubated with cell lysates and glutathione agarose beads for 30 min. Eluates were separated by SDS-PAGE gel and blotted for Rac1 (BD Biosciences) and GAPDH (Abcam). Western blots were scanned with an Odyssey imager and software (LI-COR Biosciences) and protein band intensities quantified using ImageJ; Relative Rac1 activity represents the amount of active Rac1 normalized to total Rac1 protein (Active Rac1/Total Rac1 protein).

To localize endogenous E-cadherin at cell-cell contacts, cells were plated on collagen I-coated coverslips and allowed to settle for 2.5 hours in low calcium media. Cells after time 0 were switched to normal calcium media (1.8 mM) and incubated for the indicated times at 37°C. Cells were fixed for 20 minutes in 4% paraformaldehyde, quenched in 50 mM NH_4_Cl_2_ for 10 minutes, blocked in 0.05% Saponin/10% FBS, and stained with primary and secondary antibodies for 1 hour. Cells were washed and coverslips were mounted using Aqua Polymount (Polysciences, Inc) and imaged using a Zeiss Axiovert 200 with a 100X 1.4 NA objective. Images were acquired using an AxioCam mRM camera and AxioVision Rel 4.6 software (Carl Zeiss MicroImaging).

### Rac1 Activation Assays in 3-D

Contact naïve MDCK GII cells were trypsinized, resuspended in low calcium media (5 µM), counted, 9×10^6^ cells were added to separate tubes, and centrifuged at 4°C at 900×g for 5 minutes in low calcium media (t = 0 minutes) or normal calcium media (1.8 mM) (t = 5–30 minutes) to form a 3-D cell aggregate pellet. To induce cell-cell adhesion, cell aggregates were in incubated for the indicated times at 37°C. For inhibitor studies, 9×10^6^ cells were added to separate tubes, and centrifuged at 4°C at 900×g for 5 minutes to form a cell aggregate in the presence of 10 µg/ml of monovalent DECMA-1 Fab' fragments, E-cadherin:Fc, Fc protein, or nectin2alpha:Fc in normal calcium media (1.8 mM) to induce cell-cell adhesion for the indicated times. Cells were kept in low calcium media for 35 minutes prior to switching to regular calcium media containing the various antibodies or purified fusion proteins. Isolation of active Rac1 and quantification of active Rac1 levels was performed as described above after each time point.

For imaging 3-D cell aggregates, MDCK E-cadherin-RFP stables were prepared as described above. After each time point, cell aggregates were fixed with 4% PFA for 30 min, quenched in 50 mM NH_4_Cl_2_ for 30 min and mounted in Aqua Polymount (Polysciences, Inc). To mount 3-D cell aggregates, cell pellets were flash frozen in liquid nitrogen, removed from tube, placed on glass slide with mounting media, and covered with a glass coverslip. To localize afadin, cells were blocked (PBS and 0.5% TX-100) and labeled for l-afadin (1∶100) overnight (PBS, 0.2% TX-100, 1% BSA).

Cells were imaged with a Zeiss LSM 510 Confocal Laser Scanning microscope using a Fluar 100x strain free objective, NA 1.3 (Stanford Cell Sciences Imaging Facility). Exposure time for E-cadherin fluorescence was the same for all conditions. Approximately 12-14 sections were taken at 1 µm intervals per sample. Images were projected (∼5 stacks) from the middle of the stack series using ImageJ software and images were processed and merged using Adobe Photoshop software CS2. To facilitate comparison, identical imaging and processing parameters were used within figures.

To measure E-cadherin fluorescence at cell-cell contacts, used elliptical tool on ImageJ to draw a circle (w:20×h:20, scale set to pixels). Circle was placed at cell-cell contacts (two opposing cell membranes) and the integrated density (pixel intensity) of E-cadherin-RFP at projected cell-cell contacts was quantified using ImageJ. Background measurements were taken by placing the circle in a cell-free area. Integrated density at cell-cell contacts was corrected for cell background. 50 cell-cell contacts were measured from multiple images per condition taken on the same day. For an example image of the circle drawn and what was considered background and a cell-cell contact, see Figure S2.

For Figure S2, statistical analysis was performed using the student t-test (two-tailed) in the statistical software, GraphPad Prism Version 5.0.

## Supporting Information

Figure S1 **Statistical comparison of Rac1 activity at time 0.** Bar graph shows quantification of Rac1 activation at time 0 for control, Decma Fab' fragments, E-cadherin:Fc and nectin2alpha:Fc (A). Error bars are SEM of 3 or 4 independent experiments. p-values for control vs. the various incubation conditions are displayed in table format (B).
(TIF)

Figure S2 **E-cadherin fluorescence quantification analysis example.** 1024×1024 image for each condition was placed in ImageJ and a circle was drawn and placed in a cell-free area to measure the background pixel intensity using ImageJ (arrowhead) (A). To measure E-cadherin protein intensity at cell-cell contacts, the same circle was moved to different cell-cell contacts (see arrowheads) and pixel intensity was measured using ImageJ (B). Bar: 10 µm.
(TIF)
