# Supplementary figures and images for: Rapid Suppression of Activated Rac1 by Cadherins and Nectins during De Novo Cell-Cell Adhesion
Source: PLoS One. 2011 Mar 11;6(3):e17841. doi: 10.1371/journal.pone.0017841 (PMC3055898; doi:10.1371/journal.pone.0017841)

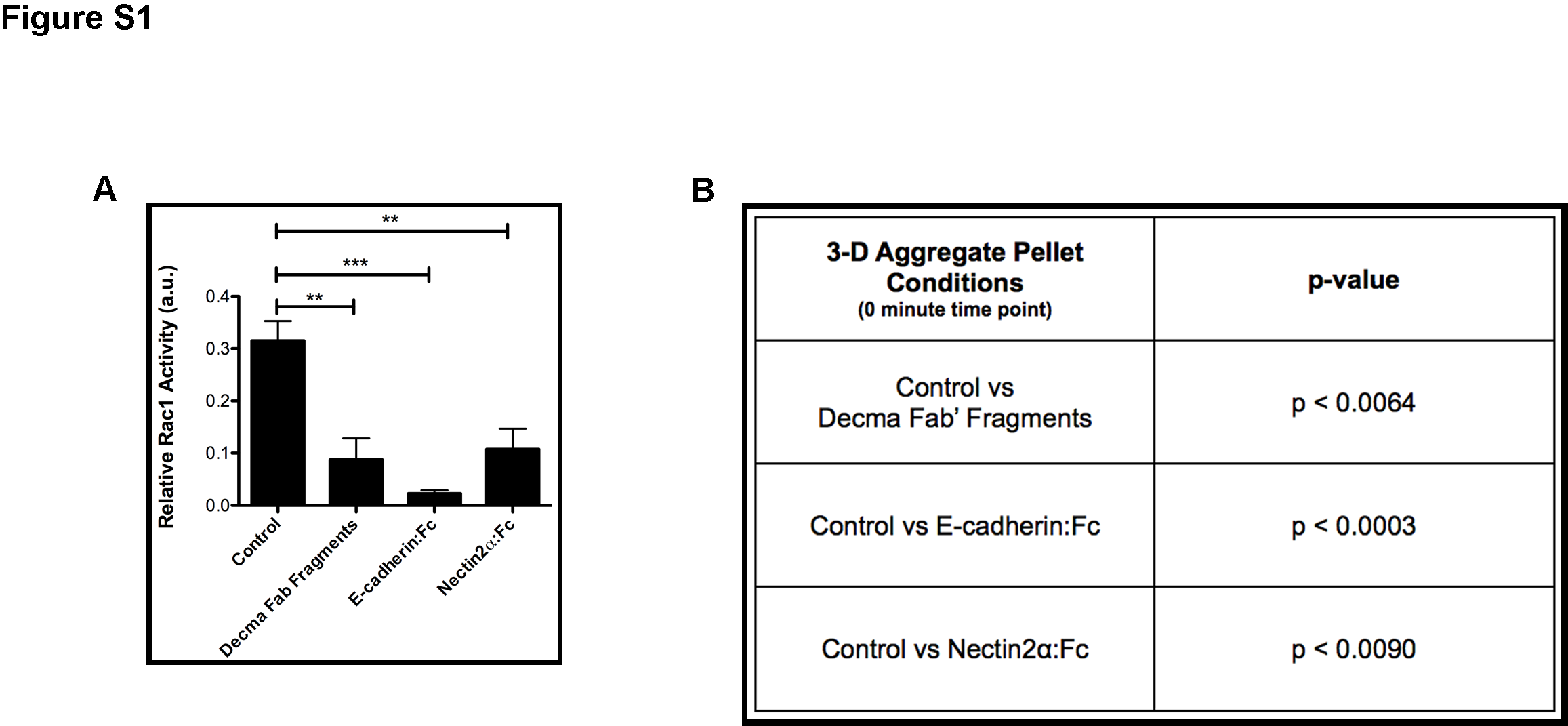

Supplement: Figure S1 — Statistical comparison of Rac1 activity at time 0. Bar graph shows quantification of Rac1 activation at time 0 for control, Decma Fab' fragments, E-cadherin:Fc and nectin2alpha:Fc (A). Error bars are SEM of 3 or 4 independent experiments. p-values for control vs. the various incubation conditions are displayed in table format (B). (TIF) [file pone.0017841.s001.tif]

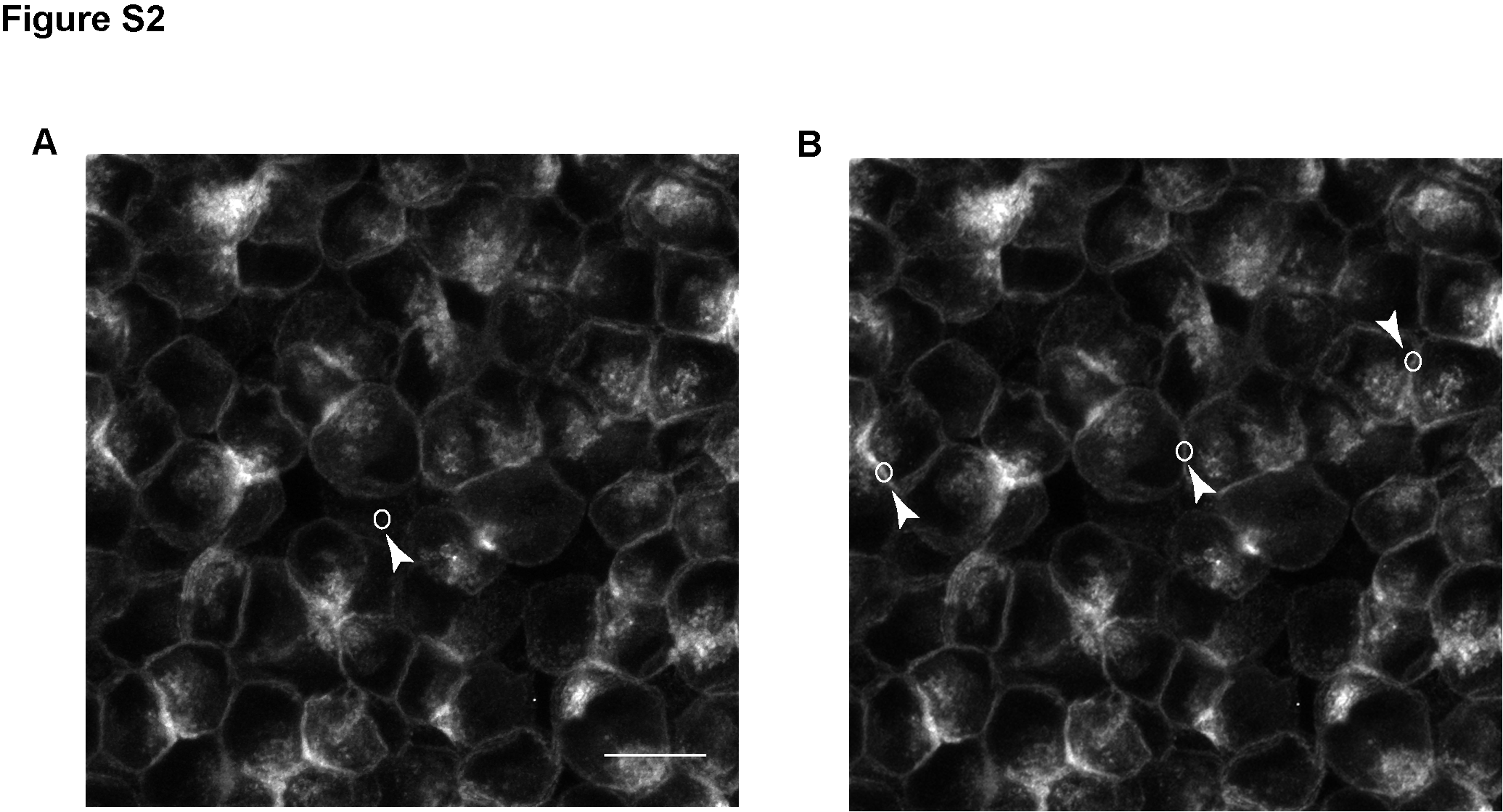

Supplement: Figure S2 — E-cadherin fluorescence quantification analysis example. 1024×1024 image for each condition was placed in ImageJ and a circle was drawn and placed in a cell-free area to measure the background pixel intensity using ImageJ (arrowhead) (A). To measure E-cadherin protein intensity at cell-cell contacts, the same circle was moved to different cell-cell contacts (see arrowheads) and pixel intensity was measured using ImageJ (B). Bar: 10 µm. (TIF) [file pone.0017841.s002.tif]
